# Retention strength of monolithic zirconia crowns cemented with different primer-cement systems

**DOI:** 10.1186/s12903-022-02223-0

**Published:** 2022-05-19

**Authors:** Mohamed Shokry, Walid Al-Zordk, Mohamed Ghazy

**Affiliations:** Department of Fixed Prosthodontics, Faculty of Dentistry, Mansoura University, Box 35516, Mansoura, Dkahlia Egypt

**Keywords:** Monolithic zirconia, Adhesive cement, Zirconia bonding, Zirconia primer, Retention test

## Abstract

**Background:**

The purpose of this in vitro study was to assess the influence of different cement systems with different ceramic primers on the retention strength of zirconia crowns.

**Methods:**

Thirty extracted molars were prepared with flat occlusal surfaces, 20 degrees taper, and 3 mm axial wall height. A zirconia crown with an occlusal bar was fabricated for each tooth. All specimens were divided (n = 10) into; Group M: Multilink Speed/Monobond N, Group P: Panavia V5/Clearfil Ceramic Primer Plus, Group D: Duo-Link universal/Z-Prime Plus. The intaglio surfaces of crowns were air-abraded using 50 µm alumina at 2.5 bar for 10 s. Then each crown was cemented onto its corresponding tooth. All specimens were thermocycled for 10,000 cycles between 5 and 55 °C. Each crown was subjected to gradually increasing vertical load along the path of insertion through hooks engaging the occlusal bar using a universal testing machine until failure. The force at dislodgment was recorded and retention strength was calculated for each specimen. The failure modes were recorded for each specimen. The data were statistically analyzed using one way ANOVA test followed by Tukey HSD test (α = .05).

**Results:**

Group D showed lowest strength (1.42 ± 0.23 MPa) and differed significantly (*P* < .001) from Group M (2.71 ± 0.45 MPa) and Group P (2.47 ± 0.41 MPa). There was no significant difference (*P* = .34) between Group M and Group P. The failure modes for Groups M and Group P were mainly cohesive, while Group D showed adhesive failure.

**Conclusions:**

The retention strength of zirconia crowns was improved with Multilink Speed and Panavia V5 cement systems, while the use of the Duo-Link Universal cement system only showed half of those retention strength values.

## Background

The use of monolithic zirconia restorations has gained popularity because of its good biocompatibility, improved esthetic quality, high strength and fracture toughness, optical properties, and the overcoming of veneer cracking or chipping problems with veneered zirconia and fabrication of the restoration from presintered homogenous blocks with CAD/CAM technique that contributes to limited amounts of manufacturing defects and reduced production time and cost [1–5]. In contrast to lithium disilicate, zirconium oxide has high surface stability and resistance to etching with hydrofluoric acid due to the lack of a glassy matrix and the inability to benefit from silane due to the lack of silicon dioxide content [6–9]. Zirconia restorations can be cemented conventionally, but the bond of zirconia to the tooth could improve the marginal seal, retention and, fracture resistance of the restored tooth and transferring of stress from ceramic to the tooth which will prevent crack initiation [10–15]. The improvement of the bonding between resin cement and zirconia can be achieved with various techniques such as airborne-particle abrasion with alumina, silica deposition methods, plasma spraying, selective infiltration etching, and application of MDP (methacryloyloxydecyl dihydrogen phosphate) primer [8, 16, 17]. Airborne-particle abrasion with 50 μm alumina particles combined with the use of an MDP-containing primer followed by a resin cement has been advocated for zirconia bonding [10, 18]. The advantages of airborne particle abrasion with aluminum oxide particles are the roughening of the zirconia which leads to a micromechanical interlocking between resin cement and zirconia as well as an increase in the surface energy and the surface area [19–21]. In addition, the advantage of using a primer containing MDP offers a chemical bonding between the acidic groups of the monomer and the oxide layer of zirconia [22–26].

The MDP monomer could be incorporated directly into various components of the cement system such as primers, adhesives, and resin cements [27]. The MDP molecule consists of methacrylate group that polymerizes into resin and has a hydrophobic group that resists hydrolysis and degradation through water uptake, and a hydrophilic group that reacts with zirconia [28, 29]. Certain ceramic primers contain a phosphate ester group and a methacrylate group and acts as an adhesive promotor that bonds to zirconia resulting in durable bond under clinical conditions [30, 31].

The most commonly used laboratory tests to measure bond strength are shear, tensile, microtensile, push out and crown retention tests [32]. The variability in outcomes between these test procedures makes it difficult to establish a correlation between laboratory data and the clinical behavior of materials tested [33]. The crown retention test is a more clinically related test for experimentation of the retention of cemented restoration [34]. In many published studies, the crown retention test has been used to assess the retention strength of cemented zirconia crowns [35–38].

The purpose of this in vitro investigation was to assess the influence of primer-cement systems with different functional phosphate monomers (Multilink Speed/Monobond N, Panavia V5/Clearfil Ceramic Primer Plus, and Duo-Link universal/Z-Prime Plus) on retention of zirconia crowns. The null hypothesis tested was that the monolithic zirconia crowns would not have similar retentive strength when cemented with different primer-cement systems with different functional phosphate monomers.

## Methods

### Specimen collection

Thirty extracted mandibular second molars with approximate dimensions, free from caries, cracks, or fractures, were collected for the current study. Approval to use human teeth was obtained from the local Research Ethics Committee. The selected teeth were cleaned and stored in a 0.1% thymol solution for 6 months at room temperature. Based on the type of cement system, the teeth were randomly assigned into (n = 10): Group M; Multilink Speed/Monobond N, Group P; Panavia V5/Clearfil Ceramic Primer Plus, Group D; Duo-Link universal/Z-Prime Plus (Table 1).

**Table 1:** Primer-cement systems used in the study (composition obtained from the manufacturer)

Primer/cement	Composition	Manufacturer
*Multilink system*		
Monobond N	MDP, 3-TMSPMA, Sulfide methacrylate, Ethanol	Ivoclar Vivadent AG, Schaan, Liechtenstein
Multilink speed	Monomer: Dimethacrylates and acidic monomers Fillers: Barium glass, ytterbium trifluoride, co-polymer and highly dispersed silicon dioxide. Initiators, stabilizers and color pigments	
*Panavia system*		
Clearfil ceramic primer plus	3-MPS, 10-MDP, Ethanol	Kuraray Dental-Kurashiki, Okayama, Japan
Panavia V5 tooth primer	10-MDP, HEMA, hydrophilic aliphatic dimethacrylate, Accelerators, Water	
Panavia V5 paste	Bis-GMA, TEGDMA, Silanated barium Glass, Colloidal silica, Surface treated aluminum oxide filler, Hydrophobic aromatic dimethacrylate, Hydrophilic aliphatic dimethacrylate, dl-Camphorquinone, Initiators, Accelerators, Pigments	
*Duo-link system*		
Z-prime plus	BPDM, HEMA, MDP, Ethanol	Bisco Inc. Schaumburg, IL, USA
All-bond universal	10-MDP, Bis-GMA, HEMA, Ethanol, Water, Initiators	
Duo-link universal	Base: Bis-GMA, TEGDMA, Glass Catalyst: Bis-GMA, TEGDMA, Glass	

*MDP* methacryloyloxydecyl dihydrogen phosphate, *TMSPMA* trimethoxysilylpropyl methacrylate, *MPS* methacryloxypropyltrimethoxysilane, *HEMA* hydroxyethyl methacrylate, *Bis-GMA* bisphenol A diglycidyl-methacrylate, *TEGDMA* triethylene glycol dimethacrylate, *BPDM* biphenyl demethacrylate

### Tooth preparation

**Fig. 1:**
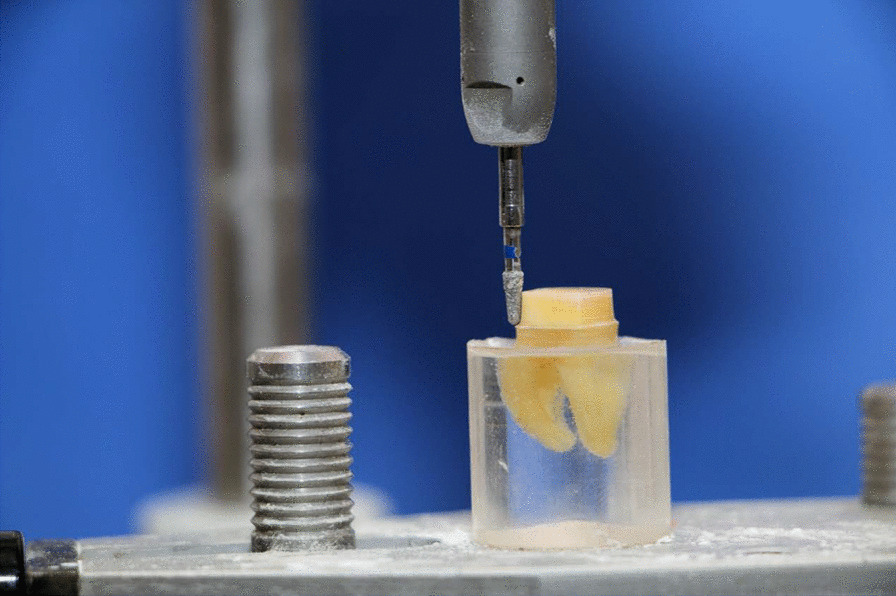
Axial walls preparation of mounted tooth using taper with rounded end diamond bur

The root of each tooth was fixed in an auto polymerizing resin material (Kemapoxy 150, CMB International, Egypt). A silicon index (Presigume Putty, President Dental, Germany) was then fabricated for each tooth. Tooth preparation was carried out with a straight handpiece (Strong Traus AT-II, Saeshin Precision Co., Korea) and a paralleling device (Marathon-103 surveyor, Saeyang Co., Korea). All preparations were done by a single operator (M.S). The height of the preparation was 3 mm with a flat occlusal surface and axial surfaces were prepared with a tapered diamond (TR-14-198/022, Mani Inc., Japan) to create 10 degrees taper with a 0.5 mm chamfer finish line [37, 39, 40] (Fig. 1).

### Determination of preparation surface area

**Fig. 2:**
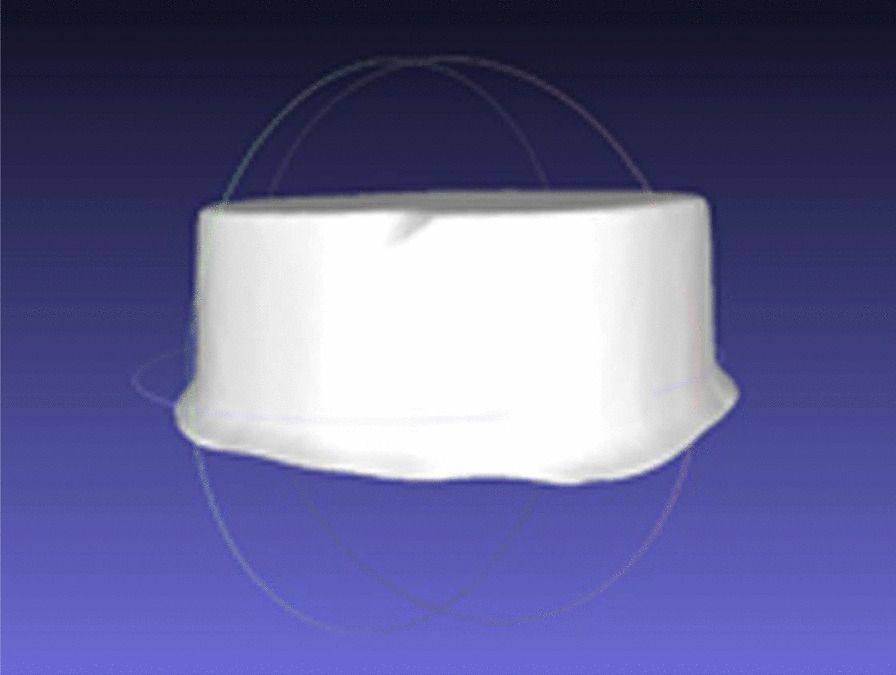
Calculation of the surface area on the 3D model of the preparation

To determine the surface area, each prepared tooth was scanned (Identica Hyprid, MEDIT Corp, Seoul, Korea). The surface area of each preparation was then calculated from the standard tessellation language (STL) file using three-dimensional computer graphics software (Meshlab software version:2020.09, Istituto di Scienzae Tecnologie dell’Informazione, Italy) (Fig. 2).

### Fabrication of the restoration

**Fig. 3:**
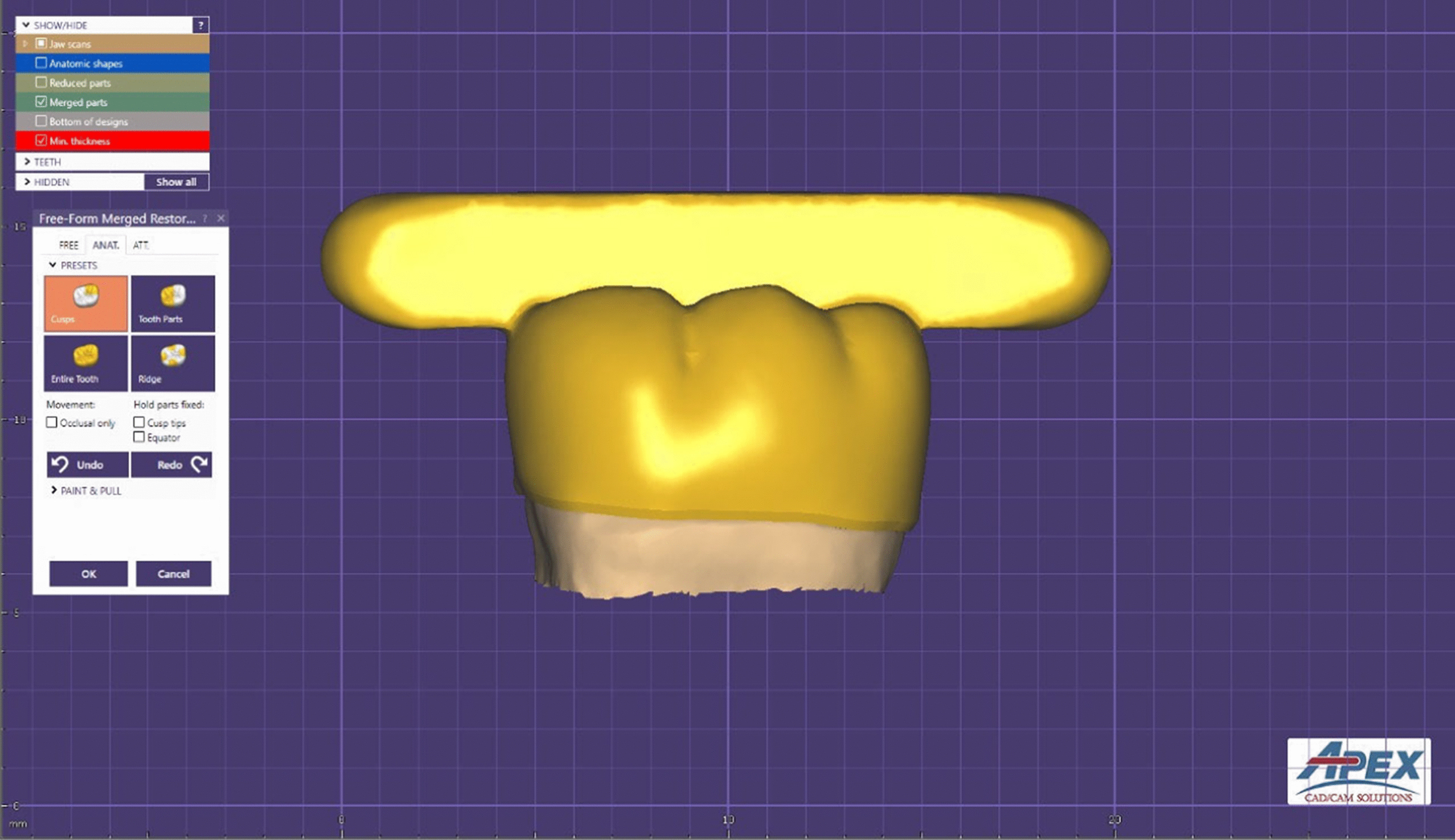
Buccal view of virtual crown on the corresponding virtual 3D abutment model with an occlusal bar

After each tooth has been scanned, the restoration was designed (collab 2017; Exocad GmbH, Germany) with an occlusal bar (15 mm × 3.5 mm × 3.5 mm) to enable vertical attachment of the cemented crown during the retention testing (Fig. 3). The bar was used to provide attachment for vertically lifting the cemented crown off the prepared tooth. All restorations were milled from a zirconia block (Katana zirconia STML A2, T14, Kuraray Dental, Japan) using a milling machine (Cori Tec 250i, imes-icore GmbH, Germany). All milled crowns were then sintered (Tabeo-100, Mihm-Vogt, Germany) following the instructions of the manufacturer.

### Cementation

The intaglio surfaces of all crowns were airborne particle abraded using 50 μm aluminum oxide particles (Cobra aluminum oxide No. 1594-1205 50 μm [270 mesh], white 5 kg canister, Renfert, Germany) under a pressure of 2.5 bar at a distance of 10 mm [30, 41]. In Group M, crowns were treated by applying a thin coat of the ceramic primer (Monobond N primer; (Ivoclar Vivadent AG, Schaan, Liechtenstein) and left for 60 s, then sprayed with air. The desired amount of cement paste (Multilink Speed; (Ivoclar Vivadent AG, Schaan, Liechtenstein) was then applied to the intaglio surface of the restoration. The crown was seated on the corresponding tooth and subjected to a static load of 40 N for 5 min using a universal testing machine (Model 3345; Instron Corp) [42]. The crown was then light cured (Bluephase Style; Ivoclar Vivadent AG, Austria) for 20 s from each surface. For Group P, the prepared tooth surfaces were treated with the tooth primer (Panavia V5 tooth primer; (Kuraray Dental-Kurashiki, Okayama, Japan) for 20 s and then gently air dried. The intaglio surfaces of restorations were treated with the ceramic primer (Clearfil Ceramic Primer Plus; (Kuraray Dental-Kurashiki, Okayama, Japan) for 10 s and gently air dried for 5 s. The cement past (Panavia V5 cement paste; (Kuraray Dental-Kurashiki, Okayama, Japan) was dispensed from its automix syringe into pretreated crowns, which were then seated and kept under static load and then light cured following the instructions of the manufacturer. For Group D, the tooth primer (All-Bond Universal; (Bisco Inc. Schaumburg, IL, USA) was applied to the prepared teeth for 20 s and sprayed with 20 s, then light cured for 20 s. The intaglio surfaces of crowns were treated with the ceramic primer (Z-Prime Plus; (Bisco Inc. Schaumburg, IL, USA) and air dried. The cement paste (Duo-Link universal cement paste; (Bisco Inc. Schaumburg, IL, USA) was then dispensed from the automix syringe into the pretreated crowns, which were then seated and kept under static load and then light cured based on the recommendations of the manufacturer.

### Aging

All specimens were thermocycled (SD Mechatronic thermocycler THE-1100, SD Mechatronics, Westerham, Germany) for 10,000 cycles between 5 and 55 °C each with a dwell time of 30 s.

### Retention test

**Fig. 4:**
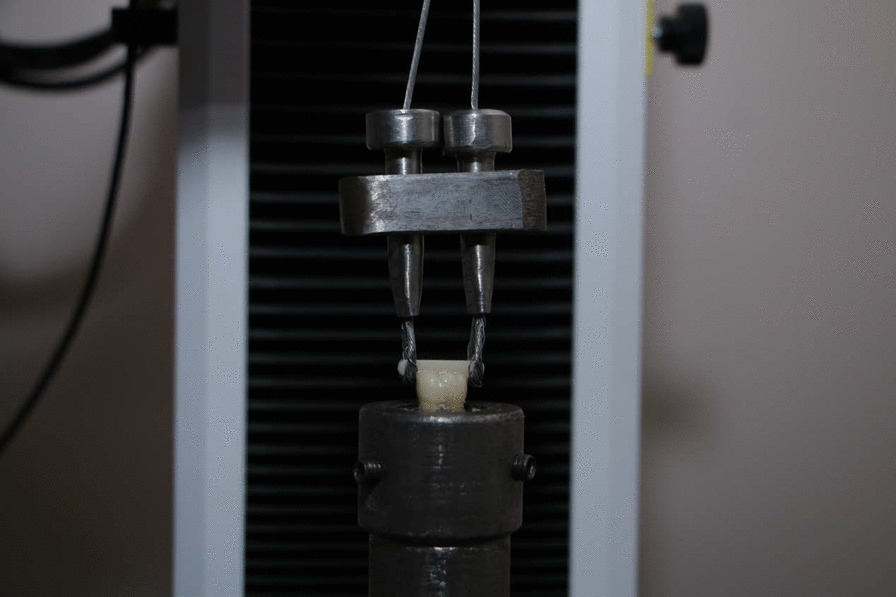
Custom made double loop made of gear wire hooked around the occlusal bar of the crown

Each specimen was secured with tightening screws to the lower fixed part of a universal testing machine (Instron, 2519-104, 3345, Canton, MA, USA). The crown was attached to the upper movable part with a custom- made loop wire that was hooked around the occlusal bar (Fig. 4). The cemented crowns were submitted to a rising vertical load along the path of insertion at a crosshead speed of 0.5 mm/min till failure occur. The force at dislodgment was noted in Newton (N) and the retention strength was calculated and recorded in Mega Pascal (MPa).

### Failure analysis

The mode of failure was classified according to cement location after removal of the crown: Class 1; adhesive failure (cement mainly remains on the prepared tooth surface), Class 2; mixed adhesive and cohesive failure (cement on crown and tooth), Class 3; adhesive failure (cement mainly the on crown), Class 4: cohesive failure (zirconia crown fracture), or Class 5; cohesive failure (tooth fracture) [35]. Selected specimens were inspected using a scanning electron microscope (JSM-6510LV, JEOl Ltd., Tokyo, Japan).

### Statistical analysis

The data were resolved using a statistical software program (SPSS v26.0; SPSS Inc). Normality of the data was tested using the Shapiro–Wilk test. A one-way ANOVA test was utilized to identify the presence of a significant difference between the mean strength values of the studied groups, followed by the Tukey HSD test (α = 0.05). Differences among failure mode patterns were identified using the Monte Carlo test.

## Results

The maximum retention load, preparation surface area, and crown retention strength values are displayed in Table 2. The highest mean retention strength value was observed in Group M (2.71 ± 0.45 MPa), followed by Group P (2.47 ± 0.41 MPa). The lowest strength value was observed for Group D (1.42 ± 0.23 MPa).

**Table 2:** Mean and standard deviation values of maximum retentive load (N), preparation surface area (mm^2^), and retention strengths (MPa) of studied groups

	Retentive load (N)	Surface area (mm^2^)	Retention strength (MPa)
Group M	422.00 ± 78.48^A^	155.61 ± 16.28	2.71 ± 0.45^C^
Group P	390.95 ± 50.43^B^	158.10 ± 13.91	2.47 ± 0.41^D^
Group D	216.85 ± 67.44^A,B^	152.91 ± 8.76	1.42 ± 0.23^C,D^

Group M: Multilink Speed/Monobond N

Group P: Panavia V5/Clearfil Ceramic Primer Plus

Group D: Duo-Link universal/Z-Prime Plus

Upper case similar letters denote significant difference

One-way ANOVA test showed significant differences in the maximum retention load (F = 27.68, *P* < 0.001) and strength (F = 33.34, *P* < 0.001) among the test groups (Table 2). The Post-hoc test showed that the retention strength of crowns cemented using Duo-Link cement system were significantly lower (*P* < 0.001) than those cemented using either Multilink or Panavia V5 cement systems. However, there was no significant difference between Group M and Group P (*P* = 0.34). There was no significant difference in prepared surface areas among the different groups (F = 0.37, *P* = 0.68).

The frequency of failure modes between study groups are presented in Table 3. The Monte Carlo test showed a significant difference in failure modes (*P* < 0.001) among the test groups. The predominant failure mode observed was cohesive within Groups M and Group P, while Group D had mainly adhesive failures (Fig. 3).

**Table 3:** Comparison of failure frequency between different studied groups (No-%)

	Class 1	Class 2	Class 3	Class 4	Class 5	Sig
Group M (n = 10)	0(0%)	3(30%)	2(20%)	3(30%)	2(20%)	*P* < 0.001
Group P (n = 10)	4(40%)	5(50%)	1(10%)	0(0%)	0(0%)	
Group D (n = 10)	4(40%)	2(20%)	4(40%)	0(0%)	0(0%)	

Class 1: Adhesive failure (cement principally on prepared dentin > 3/4 of axial surface)

Class 2: Mixed adhesive and cohesive failure (cement on crown intaglio and prepared dentin)

Class 3: Adhesive failure (cement principally on crown; > 3/4 of the intaglio)

Class 4: Cohesive failure (zirconia crown fracture)

Class 5: Cohesive failure (tooth fracture)

## Discussion

The results of the present study favor acceptance of the null hypothesis, since the retention strength of zirconia crowns zirconia was significantly affected by the type of cement system with different primers.

In the current study, monolithic zirconia was used because of its high flexural strength and fracture toughness and can be used in thickness as low as 0.5 mm [3–5]. In contrast to glass ceramic, however, zirconia is not susceptible to etching because it does not contain a glassy matrix which rules out the use of conventional adhesive procedures [6]. One self adhesive resin cement (Multilink Speed) and two self etch adhesive resin cements (Panavia V5 and Duo-Link universal) were selected for the current study. These cements were chosen for their good physical properties and ease of use [7]. The resin cements containing the MDP monomer can lead to a high bond strength to zirconia due because of reaction between the hydroxyl groups of the zirconia surface and the phosphate ester group of the MDP molecules [11, 15, 17]. The acidic monomers of the self adhesive resin cement can wet and bond to the exposed hydrophilic inorganic fillers that are created by air abrasion of the zirconia surface [12]. In addition, Bis-GMA is a usually embloyed crosslinking monomer in self etch adhesive resin cements systems which bonds strongly to dentin by forming a hybrid layer with an uncovered dentin collagen fibril [8].

The crown retention test was used in this study because it simulates clinical use for testing the retention strength of resin cements and the results are reproducible and less variable [32, 34, 37]. In this study, compromised preparation criteria was performed for the retention test with a flat occlusal surface, 3 mm preparation height, and a high angle of convergence 20 degrees [32, 39]. These criteria can help ensure that crowns are depend primarily on cementation rather than the geometry of tooth preparation and avoid tooth fracture which is an unwanted situation [39]. The aim of retention test was to evaluate the retentive quality of the resin cement rather than the cohesive strength of the tooth [32].

The outcomes of the present investigation showed that the highest retention strength value was recorded for Group M and the least retention strength value was recorded for Group D. This finding is supported by previous studies in which MDP containing primers with air abraded zirconia surfaces were used to enhance physicochemical interaction between zirconia and the resin cement [6, 23–25, 30]. In addition to a functional phosphoric acid group in the MDP molecule, the ceramic primer (Monobond N) also contains methacrylate monomers, which together can form a stable bond that is resistant to hydrolysis and offers a strong and durable bond with zirconium oxide [15, 20]. Amaral et al. [29], showed higher bond strengths when using the Monobond Plus ceramic primer.

There was no significant difference in retention strength between Groups M and Group P in this investigation. The Panavia V5 tooth primer contains co-initiators that initiate the conversion with an initiator of the resin cement on contact without light curing, which leads to good polymerization of resin cement paste and offers superior dentin bonding performance [43]. Furthermore, owing of its low water sorption, Panavia V5 resin cement is less influenced by thermal cycling [13]. Furthermore, Clearfil Ceramic Primer Plus contains MDP and a silane intended to increase chemical bonding to air abraded zirconia [14]. A previous study showed that treating zirconia with Clearfil Ceramic Primer or Monobond Plus gave the best results. In addition, other studies have found higher a bond strength for Panavia V5 resin cement [9, 21].

Group D had the lowest retention strength, which could be elucidated by the fact that Duo-link resin cement is a Bis-GMA based cement, and Bis-GMA based resin cement has been shown to have lower adhesive strength to zirconia than adhesive phosphate monomer-based resin cements [39]. Also, Z-prime plus includes two adhesive monomers (carboxylate and MDP), and the presence of carboxylic acid monomers can weaken the bond between this primer and methacrylate groups found in this resin cement [26]. It was assumed that the lower bond strength of Duo-link and Z Prime plus is due to chemical differences in the base monomers or solvents of the primers, differences in the primer initiation systems, or to variations in the concentration of MDP [17]. Furthermore, dentin bonding agent can be classified as total etch, one step self etch and self etching primer/adhesive system, also may be HEMA based or HEMA free and All Bond Universal is a HEMA based dentin bonding agent which may be susceptible to water uptake from dentinal tubules and permeate polymerized hydrophilic bonding agent as a result it may interfere with subsequent coupling with Duo-link Universal [44].

The mode of failure for Group M in this investigation was largely cohesive. The cohesive failures were most often observed within the resin cement in Group P. Such failure indicates high bond strength, as it is assumed that the bond strength to the crown and dentin surfaces is higher than the tensile strength of the cement. Halabi et al. [14] showed a predominantly cohesive failure within the Panavia V5 resin cement compared to adhesive failures at the interface of resin and dentin of Estecem II and Rely X Ultimate. The adhesive failures were observed in Group D. This result is in line with another study that compares the adhesive failure mode of Duo-Link Universal to the cohesive failure in Rely X Unicem and Panavia F2.0 [39].

**Fig. 5:**
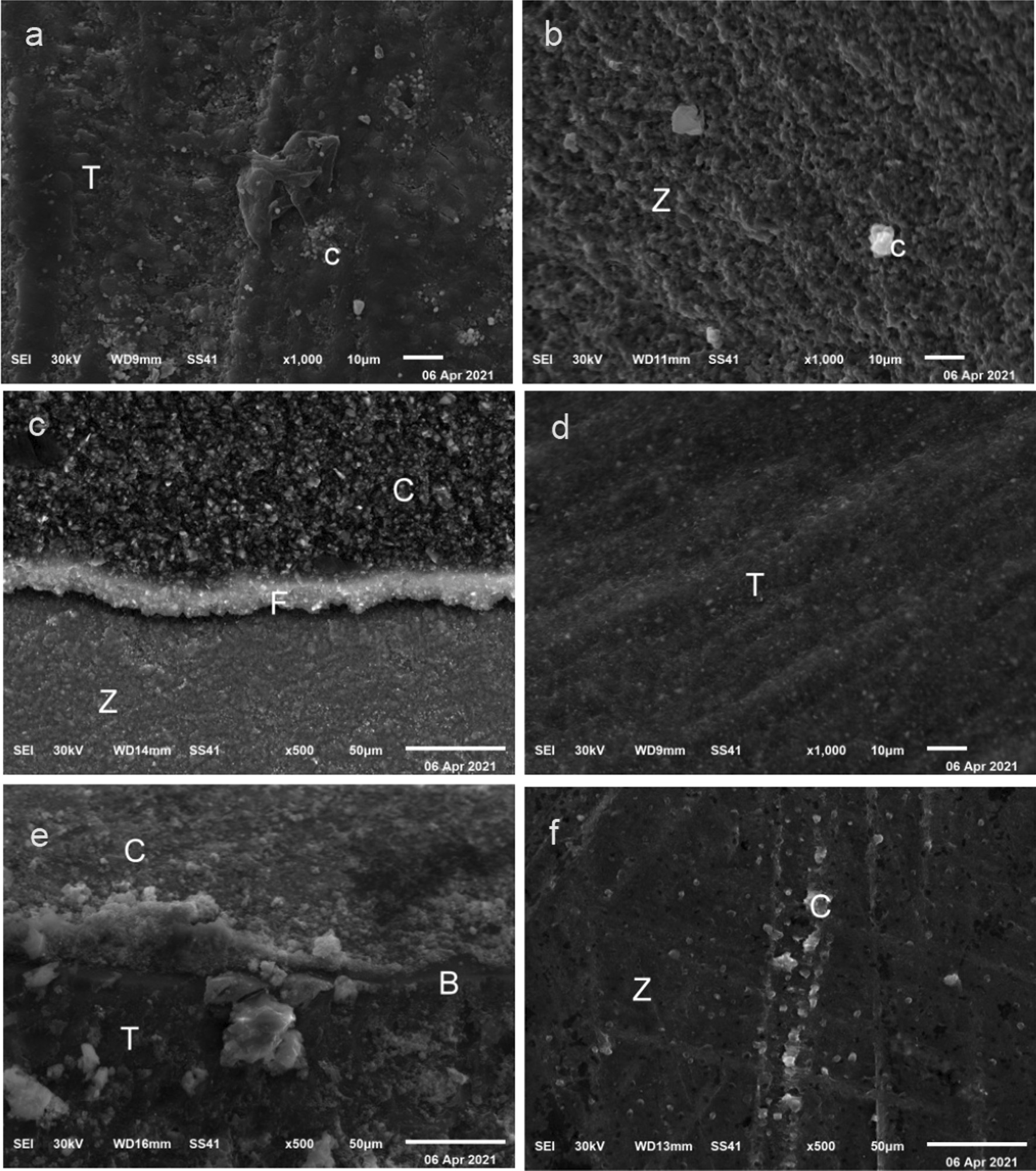
SEM photomicrographs of representative fractured specimens; **a** occlusal surface of the tooth with cement fragments from Group M, **b** zirconia surface with cement particles from Group M, **c** fractured surface at the interface between zirconia and cement from Group P, **d** tooth surface without any cement remnants from Group D, **e**: fractured surface at the interface between zirconia and cement from Group D, **f** air abraded zirconia surface with cement particles from Group D. T: tooth, Z: zirconia, F: interface between zirconia and cement

Scanning electron microscope examination of the zirconia surfaces for Groups M and Group P showed a preponderance of residual resin cement that remained on the zirconia surfaces [14, 15]. Specimens from Group D showed a large proportion of resin cement that remained on tooth surfaces [39] (Fig. 5).

Limitations of this in vitro study were the lack of load aging, various preparation heights, different convergence angles, and different crown thicknesses. The current study used thermal cycling to mimic the stresses of an intraoral environment but used water baths instead of artificial saliva. Also, only one type of zirconia was used and the results cannot be generalized to other zirconia materials.

## Conclusions

Within the limitations of this in vitro study, it was concluded that;

The retention strength of zirconia crowns was improved with Multilink Speed and Panavia V5 cement systems, while the use of the Duo-Link Universal cement system only showed half of those retention strength values.

## Data Availability

The datasets used and/or analyzed during the current study available from the corresponding author on reasonable request.

## Abbreviations

MDP: Methacryloyloxydecyl dihydrogen phosphate
TMSPMA: Trimethoxysilylpropyl methacrylate
MPS: Methacryloxypropyltrimethoxysilane
HEMA: Hydroxyethyl methacrylate
Bis-GMA: Bisphenol A diglycidyl-methacrylate
TEGDMA: Triethylene glycol dimethacrylate
BPDM: Biphenyl demethacrylate

## References

[1] Shiraishi T, Watanabe I (2016). Thickness dependence of light transmittance, translucency and opalescence of a ceria-stabilized zirconia/alumina nanocomposite for dental applications. Dent Mater.

[2] Nordahl N, von Steyern PV, Larsson C (2015). Fracture strength of ceramic monolithic crown systems of different thickness. J Oral Sci.

[3] Zhang Y, Lawn B (2018). Novel zirconia materials in dentistry. J Dent Res.

[4] Harada K, Raigrodski AJ, Chung KH, Flinn BD, Dogan S, Mancl LA (2016). A comparative evaluation of the translucency of zirconias and lithium disilicate for monolithic restorations. J Prosthet Dent.

[5] Kim HK, Kim SH, Lee JB, Han JS, Yeo IS, Ha SR (2016). Effect of the amount of thickness reduction on color and translucency of dental monolithic zirconia ceramics. J Adv Prosthodont.

[6] Chuang SF, Kang LL, Liu YC, Lin JC, Wang CC, Chen H-M (2017). Effects of silane and MDP-based primers application orders on zirconia–resin adhesion—A ToF-SIMS study. Dent Mater.

[7] Passos SP, May LG, Barca DC, Özcan M, Bottino MA, Valandro LF (2010). Adhesive quality of self-adhesive and conventional adhesive resin cement to Y-TZP ceramic before and after aging conditions. Oper Dent.

[8] van Vuuren WAJ, Wong P, Al-Amleh B, Lyons KM, Chun Li K, Waddell JN (2018). Effect of a glaze layer on adhesion energy between resin cements to zirconia ceramic. Int J Adhes Adhes.

[9] Valente F, Mavriqi L, Traini T (2020). Effects of 10-MDP based primer on shear bond strength between zirconia and new experimental resin cement. Materials (Basel).

[10] Lima RBW, Barreto SC, Alfrisany NM, Porto TS, De Souza GM, De Goes MF (2019). Effect of silane and MDP-based primers on physico-chemical properties of zirconia and its bond strength to resin cement. Dent Mater.

[11] Lin J, Shinya AA, Gomi H, Shinya AA (2010). Effect of self-adhesive resin cement and tribochemical treatment on bond strength to zirconia. Int J Oral Sci.

[12] Hampe R, Keller M, Roos M, Herrero P, Stawarczyka B (2018). Effect of conditioning agents combined with two adhesive resin cements on micro-Tensile Bond Strength to polymeric CAD/CAM materials. Int J Adhes Adhes.

[13] Müller JA, Rohr N, Fischer J (2017). Evaluation of ISO 4049: water sorption and water solubility of resin cements. Eur J Oral Sci.

[14] Halabi S, Sato T, Ikeda M, Nikaido T, Burrow MF, Tagami J (2019). Adhesion durability of dualcure resin cements and acid–base resistant zone formation on human dentin. Dent Mater.

[15] Elsaka SE (2016). Influence of surface treatment on the bond strength of resin cements to monolithic zirconia. J Adhes Dent.

[16] Franz A, Winkler O, Lettner S, Öppinger S, Hauser A, Haidar M, Moritz A, Watts DC (2021). Optimizing the fitting-surface preparation of zirconia restorations for bonding to dentin. Dent Mater.

[17] Pitta J, Branco TC, Portugal J (2018). Effect of saliva contamination and artificial aging on different primer/cement systems bonded to zirconia. J Prosthet Dent.

[18] Sadighpour L, Geramipanah F, Fazel A, Allahdadi M, Kharazifard MJ (2018). Effect of selected luting agents on the retention of CAD/CAM zirconia crowns under cyclic environmental pressure. J Dent (Tehran).

[19] Aleisa K, Alwazzan K, Al-Dwairi ZN, Almoharib H, Alshabib A, Aleid A (2013). Retention of zirconium oxide copings using different types of luting agents. J Dent Sci.

[20] Steiner R, Heiss-Kisielewsky I, Schwarz V, Schnabl D, Dumfahrt H, Laimer J, Steinmassl O, Steinmassl PA (2019). Zirconia primers improve the shear bond strength of dental zirconia. J Prosthodont.

[21] Inokoshi M, Poitevin A, De Munck J, Minakuchi S, Van Meerbeek B (2014). Bonding effectiveness to different chemically pre-treated dental zirconia. Clin Oral Investig.

[22] de Souza GMD, Thompson VP, Braga RR (2011). Effect of metal primers on microtensile bond strength between zirconia and resin cements. J Prosthet Dent.

[23] Takagaki T, Lyann SK, Ikeda M, Inokoshi M, Sadr A, Nikaido T (2019). Effects of aluminablasting pressure on the bonding to super/ultra-translucent zirconia. Dent Mater.

[24] Chen C, Chen Y, Lu Z, Qian M, Xie H, Tay FR (2017). The effects of water on degradation of the zirconia-resin bond. J Dent.

[25] Özcan M, Bernasconi M (2015). Adhesion to zirconia used for dental restorations: a systematic review and meta-analysis. J Adhes Dent.

[26] Fernando A, Marina S, Carlos D, Walter G, Paulo F (2014). Association of different primers and resin cements for adhesive bonding to zirconia ceramics. J Adhes Dent.

[27] de Souza G, Hennig D, Aggarwal A, Tam LE (2014). The use of MDP-based materials for bonding to zirconia. J Prosthet Dent.

[28] Yi YA, Ahn JS, Park YJ, Jun SH, Lee IB, Cho BH, Son HH, Seo DG (2015). The effect of sandblasting and different primers on shear bond strength between yttria tetragonal zirconia polycrystal ceramic and a self- adhesive resin cement. Oper Dent.

[29] Amaral M, Beli R, Cesar PF, Valandro LF, Petschelt A, Lohbauer U (2014). The potential of novel primers and universal adhesives to bond to zirconia. J Dent.

[30] Kern M (2015). Bonding to oxide ceramics—laboratory testing versus clinical outcome. Dent Mater.

[31] Inokoshi M, Kameyama A, De Munck J, Minakuchi S, Van Meerbeek B (2013). Durable bonding to mechanically and/or chemically pre-treated dental zirconia. J Dent.

[32] Heintze S (2010). Crown pull-off test (crown retention test) to evaluate the bonding effectiveness of luting agents. Dent Mater.

[33] Heintze SD (2013). Clinical relevance of tests on bond strength, microleakage and marginal adaptation. Dent Mater.

[34] Rohr N, Brunner S, Märtin S, Fischer J (2018). Influence of cement type and ceramic primer on retention of polymer-infiltrated ceramic crowns to a one-piece zirconia implant. J Prosthet Dent.

[35] Lepe X, Streiff KR, Johnson GH (2021). Long-term retention of zirconia crowns cemented with current automixed cements. J Prosthet Dent.

[36] Maischberger C, Liebermann A, Stawarczyk B (2019). The effect of hemostatic agents on the retention strength of zirconia crowns luted to dentin abutments. Materials (Basel).

[37] Ehlers V, Kampf G, Stender E, Willershausen B, Ernst CP (2015). Effect of thermocycling with or without 1 year of water storage on retentive strengths of luting cements for zirconia crowns. J Prosthet Dent.

[38] Sellers K, Powers JM, Kiat-Amnuay S (2017). Retentive strength of implant supported CAD-CAM lithium disilicate crowns on zirconia custom abutments using 6 different cements. J Prosthet Dent.

[39] Saryazdi MK, Zadeh RS, Givan D, Burgess JO, Ramp LC, Liu PR (2014). Influence of surface treatment of yttrium-stabilized tetragonal zirconium oxides and cement type on crown retention after artificial aging. J Prosthet Dent.

[40] Winkelmeyer C, Wolfart S, Marotti J (2016). Analysis of tooth preparations for zirconia-based crowns and fixed dental prostheses using stereolithography data sets. J Prosthet Dent.

[41] Barreto SC, Lima RBW, Aguiar FHB, Santos CTD, Paulillo LA, de Souza G (2020). Mechanical properties of aged yttria-stabilized tetragonal zirconia polycrystal after abrasion with different aluminum oxide particles. J Prosthet Dent.

[42] Kale E, Yilmaz B, Seker E, Özcelik TB (2017). Effect of fabrication stages and cementation on the marginal fit of CAD-CAM monolithic zirconia crowns. J Prosthet Dent.

[43] Akehashi S, Takahashi R, Nikaido T, Burrow MF, Tagami J (2019). Enhancement of dentin bond strength of resin cement using new resin coating materials. Dent Mater J.

[44] Montanari M, Fiorillo L, Cervino G, Sambataro S, Herford AS, Cicciù M (2021). The effect of different condition of pulpal pressure on microtensile bond strength of several dentin bonding agents on deep and superficial dentin. Materials.

